# Supplementary material for: Baseline Tyrosine Level Is Associated with Dynamic Changes in FAST Score in NAFLD Patients under Lifestyle Modification
Source: Metabolites. 2023 Mar 17;13(3):444. doi: 10.3390/metabo13030444 (PMC10058052; doi:10.3390/metabo13030444)

Supplementary Information

Baseline Tyrosine Level Is Associated with Dynamic Changes in FAST™ Score in NAFLD Patients Under Lifestyle Modification

Hwi Young Kim [1,†], Da Jung Kim [2,†], Hye Ah Lee [3], Joo-Youn Cho [4,5,*] and Won Kim [6,*]

[1] Department of Internal Medicine, College of Medicine, Ewha Womans University, Seoul, Republic of Korea
[2] Metabolomics Core Facility, Department of Transdisciplinary Research and Collaboration, Biomedical Research Institute, Seoul National University Hospital, Seoul, Republic of Korea
[3] Clinical Trial Center, Ewha Womans University Medical Center, Seoul, Republic of Korea
[4] Department of Clinical Pharmacology and Therapeutics, Seoul National University College of Medicine and Hospital, Seoul, Republic of Korea; joocho@snu.ac.kr
[5] Department of Biomedical Sciences, Seoul National University College of Medicine, Seoul, Republic of Korea
[6] Department of Internal Medicine, Seoul National University College of Medicine and Seoul Metropolitan Government Boramae Medical Center, Seoul, Republic of Korea
† These two authors contributed equally to this work as co-first authors.
* These two authors contributed equally to this work as co-corresponding authors.
* Correspondence: drwon1@snu.ac.kr, Tel.: +82-2 870-2233 (WK); joocho@snu.ac.kr, Tel.: +82-2-740-8286 (JYC)

Supplementary Methods

Study Participants

The diagnosis of NAFLD was made by the presence of at least two of the following three findings on abdominal ultrasonography: (i) diffusely increased liver echogenicity ("bright") that was greater than that for kidney, (ii) vascular blurring, and (iii) deep attenuation of the ultrasound signal [33].

Baseline Clinical and Laboratory Assessments

Anthropometric measurements were recorded by a well-trained nurse according to a consistent protocol. Weight was measured to the nearest 0.1 kg, and height was measured to the nearest 0.1 cm. Body mass index (BMI) was calculated as weight (kg) divided by height squared (m^2). Waist circumference was measured at the mid-point between the highest point of the iliac crest and the last floating rib to the nearest 0.1 cm at the end of normal expiration [34].

Weight-adjusted skeletal muscle index (SMI_wt) was calculated using a modified version of the formula by Janssen et al. [35, 36], as follows: total skeletal muscle mass/weight × 100. Sarcopenia was defined as an SMI_wt beyond two standard deviations (SDs) below the gender-specific mean for healthy young adults according to nationwide health examinations of the Korean population (SMI_wt <29.0 in men or <22.9 in women) [37, 38]. The proportion of body weight consisting of fat was expressed as a percentage of the total body weight (fat%). Venous blood samples were drawn after a 12 h overnight fast, and plasma was separated immediately via centrifugation. Insulin resistance was evaluated using the homeostasis model assessment of insulin resistance (HOMA-IR) [39].

Metabolomics and Genotyping

The sample preparation and liquid chromatography with tandem mass spectrometry (LC-MS/MS) analytical procedure are described in detail in our previous reports [40, 41]. For quantitation of bile acids, plasma samples were prepared according to the manufacturer's instructions. For LC-MS/MS analyses, 10 µL of diluted filtrate was injected into an SHIMADZU Nexera (Shimadzu Corporation, Kyoto, Japan) ultra-high-performance liquid chromatography (UHPLC) system consisting of a binary pump and a thermostat set at 5 °C. Separation was achieved on an analytical column using the Biocrates® Bile Acids Kit (BIOCRATES Life Science AG, Innsbruck, Austria) equipped with an AJ0-4287 SecurityGuard™ ULTRA cartridge for C18 HPLC columns (Phenomenex, Torrance, CA). Mobile phase A comprised 10 mM ammonium acetate (NH4Ac) and 0.015% formic acid in Milli-Q® water, and mobile phase B comprised 10 mM NH4Ac and 0.015% formic acid in acetonitrile:methanol:Milli-Q® water 65:30:5 (v/v/v). A Triple Quadrupole 6500 plus system equipped with an electrospray ionization source was used for MS analysis. The 17 BAs were identified and quantified via the LC-MS/MS system (scheduled multiple reaction monitoring). The abundance of each BA was calculated from the area under the curve by normalizing to its respective isotope-labeled internal standard using Analyst® 1.5.2 software (AB Sciex, Framingham, MA). Calibration curves, quality controls, and samples were evaluated using MetIDQ™ software (BIOCRATES).

Genotyping was performed using the following: TaqMan 50-nuclease assays (Life Technologies, Carlsbad, CA) for *PNPLA3* rs738409 C>G, *TM6SF2* rs58542926 C>T, *SREBF2* rs133291 C>T, and *MBOAT7-TMC4* rs641738 C>T as well as Sanger sequencing (Macrogen, Inc., Seoul, South Korea) for *HSD17B13* rs72613567 adenine insertion (A-INS) single-nucleotide polymorphisms [42-44].

Lifestyle Modification and Follow-up Assessments

Study participants were consulted for dietary and exercise education at baseline according to practice guidelines [4,45,46]. Briefly, reduced caloric intake of more than 500 kcal/day was recommended to participants, considering their age, sex, and BMI. Each participant was also encouraged to increase physical activity by performing moderate-intensity exercise for more than 30 minutes more than 3 time/week, in conjunction with hypocaloric diet. Follow-up visits were planned for each participant every 3–6 months based on individual level of disease severity and adherence. At each follow-up visit, adherence to lifestyle modification was assessed, along with anthropometric measurements and routine laboratory tests. Follow-up VCTE was performed on a yearly basis.

Statistical Analysis

Genetic analyses were performed using an additive model (by coding the genotypes 0, 1, and 2 for wild-type homozygotes, heterozygotes, and alternate allele homozygotes, respectively), a dominant model (by coding the genotypes 0 and 1 for wild-type homozygotes and [heterozygotes + alternate allele homozygotes], respectively), or a recessive model (by coding the genotypes 0 and 1 for [wild-type homozygotes + heterozygotes] and alternate allele homozygotes, respectively).

References

4. Chalasani, N.; Younossi, Z.; Lavine, J.E.; Charlton, M.; Cusi, K.; Rinella, M.; Harrison, S.A.; Brunt, E.M.; Sanyal, A.J. The diagnosis and management of nonalcoholic fatty liver disease: Practice guidance from the American Association for the Study of Liver Diseases. *Hepatology* **2018**, *67*, 328–357, DOI:10.1002/hep.29367.

33. Farrell, G.C.; Chitturi, S.; Lau, G.K.; Sollano, J.D. Guidelines for the assessment and management of non-alcoholic fatty liver disease in the Asia-Pacific region: executive summary. *J. Gastroenterol. Hepatol.* **2007**, *22*, 775–777, DOI:10.1111/j.1440-1746.2007.05002.x.

34. World Health Organization. Obesity: preventing and managing the global epidemic. 2000.

35. Cruz-Jentoft, A.J.; Bahat, G.; Bauer, J.; Boirie, Y.; Bruyère, O.; Cederholm, T.; Cooper, C.; Landi, F.; Rolland, Y.; Sayer, A.A.; Schneider, S.M.; Sieber, C.C.; Topinkova, E.; Vandewoude, M.; Visser, M.; Zamboni, M. Sarcopenia: revised European consensus on definition and diagnosis. *Age Ageing* **2019**, *48*, 601, DOI:10.1093/ageing/afz046.

36. Buckinx, F.; Landi, F.; Cesari, M.; Fielding, R.A.; Visser, M.; Engelke, K.; Maggi, S.; Dennison, E.; Al-Daghri, N.M.; Allepaerts, S.; Bauer, J.; Bautmans, I.; Brandi, M.L.; Bruyère, O.; Cederholm, T.; Cerreta, F.; Cherubini, A.; Cooper, C.; Cruz-Jentoft, A.; McCloskey, E.; Dawson-Hughes, B.; Kaufman, J.M.; Laslop, A.; Petermans, J.; Reginster, J.Y.; Rizzoli, R.; Robinson, S.; Rolland, Y.; Rueda, R.; Vellas, B.; Kanis, J.A. Pitfalls in the measurement of muscle mass: a need for a reference standard. *J. Cachexia Sarcopenia Muscle* **2018**, *9*, 269–278, DOI:10.1002/jcsm.12268.

37. Kim, Y.S.; Lee, Y.; Chung, Y.S.; Lee, D.J.; Joo, N.S.; Hong, D.; Song, G.e.; Kim, H.J.; Choi, Y.J.; Kim, K.M. Prevalence of sarcopenia and sarcopenic obesity in the Korean population based on the Fourth Korean National Health and Nutritional Examination Surveys. *J. Gerontol. A Biol. Sci. Med. Sci.* **2012**, *67*, 1107–1113, DOI:10.1093/gerona/gls071.

38. Kim, J.H.; Lim, S.; Choi, S.H.; Kim, K.M.; Yoon, J.W.; Kim, K.W.; Lim, J.Y.; Park, K.S.; Jang, H.C. Sarcopenia: an independent predictor of mortality in community-dwelling older Korean men. *J. Gerontol. A Biol. Sci. Med. Sci.* **2014**, *69*, 1244–1252, DOI:10.1093/gerona/glu050.

39. Matthews, D.R.; Hosker, J.P.; Rudenski, A.S.; Naylor, B.A.; Treacher, D.F.; Turner, R.C. Homeostasis model assessment: insulin resistance and beta-cell function from fasting plasma glucose and insulin concentrations in man. *Diabetologia* **1985**, *28*, 412–419, DOI:10.1007/BF00280883.

40. Kim, D.J.; Cho, E.J.; Yu, K.S.; Jang, I.J.; Yoon, J.H.; Park, T.; Cho, J.Y. Comprehensive metabolomic search for biomarkers to differentiate early stage hepatocellular carcinoma from cirrhosis. *Cancers (Basel)* **2019**, *11*, 1497, DOI:10.3390/cancers11101497.

41. Kim, D.J.; Oh, J.Y.; Rhee, C.K.; Park, S.J.; Shim, J.J.; Cho, J.Y. Metabolic fingerprinting uncovers the distinction between the phenotypes of tuberculosis associated COPD and smoking-induced COPD. *Front. Med. (Lausanne)* **2021**, *8*, 619077, DOI:10.3389/fmed.2021.619077.

42. Abul-Husn, N.S.; Cheng, X.; Li, A.H.; Xin, Y.; Schurmann, C.; Stevis, P.; Liu, Y.; Kozlitina, J.; Stender, S.; Wood, G.C.; Stepanchick, A.N.; Still, M.D.; McCarthy, S.; O'Dushlaine, C.; Packer, J.S.; Balasubramanian, S.; Gosalia, N.; Esopi, D.; Kim, S.Y.; Mukherjee, S.; Lopez, A.E.; Fuller, E.D.; Penn, J.; Chu, X.; Luo, J.Z.; Mirshahi, U.L.; Carey, D.J.; Still, C.D.; Feldman, M.D.; Small, A.; Damrauer, S.M.; Rader, D.J.; Zambrowicz, B.; Olson, W.; Murphy, A.J.; Borecki, I.B.; Shuldiner, A.R.; Reid, J.G.; Overton, J.D.; Yancopoulos, G.D.; Hobbs, H.H.; Cohen, J.C.; Gottesman, O.; Teslovich, T.M.; Baras, A.; Mirshahi, T.; Gromada, J.; Dewey, F.E. A protein-truncating HSD17B13 variant and protection from chronic liver disease. *N. Engl. J. Med.* **2018**, *378*, 1096–1106, DOI:10.1056/NEJMoa1712191.

43. Pirola, C.J.; Garaycoechea, M.; Flichman, D.; Arrese, M.; San Martino, J.; Gazzi, C.; Castaño, G.O.; Sookoian, S. Splice variant rs72613567 prevents worst histologic outcomes in patients with nonalcoholic fatty liver disease. *J. Lipid Res.* **2019**, *60*, 176–185, DOI:10.1194/jlr.P089953.

44. Koo, B.K.; Joo, S.K.; Kim, D.; Lee, S.; Bae, J.M.; Park, J.H.; Kim, J.H.; Chang, M.S.; Kim, W. Development and validation of a scoring system, based on genetic and clinical factors, to determine risk of steatohepatitis in Asian patients with nonalcoholic fatty liver disease. *Clin. Gastroenterol. Hepatol.* **2020**, *18*, 2592–2599.e10, DOI:10.1016/j.cgh.2020.02.011.

45. Marchesini, G.; Day, C.h.P.; Dufour, J.F.; Canbay, A.; Nobili, V.; Ratziu, V.; Tilg, H.; Roden, M.; Gastaldelli, A.; Yki-Järvinen, H.; Schick, F.; Vettor, R.; Frühbeck, G.; Mathus-Vliegen, L. EASL-

EASD-EASO clinical practice guidelines for the management of non-alcoholic fatty liver disease. *J. Hepatol.* **2016**, *64*, 1388–1402, DOI:10.1016/j.jhep.2015.11.004.
46. Kang, S.H.; Lee, H.W.; Yoo, J.J.; Cho, Y.; Kim, S.U.; Lee, T.H.; Jang, B.K.; Kim, S.G.; Ahn, S.B.; Kim, H.; Jun, D.W.; Choi, J.I.; Song, D.S.; Kim, W.; Jeong, S.W.; Kim, M.Y.; Koh, H.; Jeong, S.; Lee, J.W.; Cho, Y.K. KASL clinical practice guidelines: Management of nonalcoholic fatty liver disease. *Clin. Mol. Hepatol.* **2021**, *27*, 363–401, DOI:10.3350/cmh.2021.0178.

Supplementary Tables

Table S1. Longitudinal changes in patient distribution according to the FAST score at baseline and at follow-up.

		At follow–up		
		FAST ≤0.35	0.35<FAST<0.67	FAST ≥0.67
At baseline	FAST ≤0.35	79	5	0
	0.35<FAST<0.67	43	15	5
	FAST ≥0.67	4	6	3

Abbreviations: FAST, Fibroscan-aspartate aminotransferase

Table S2. Changes in parameters according to weight change during follow-up.

	Weight loss >5% (n=30, 18.8%)		Weight loss ≤5% (n=75, 46.9%)		Weight gain (n=55, 34.4%)		P	P for post–hoc test		
	Adjusted mean	95% CI	Adjusted mean	95% CI	Adjusted mean	95% CI		Wt. loss ≥5% vs. <5%	Wt. loss <5% vs. Wt. gain	Wt. loss ≥5% vs. Wt. gain
ΔBwt	−6.96	−8.09, −5.82	−1.70	−2.42, −0.99	2.78	1.94, 3.61	<0.001	<0.001	<0.001	<0.001
ΔBMI	−2.85	−3.53, −2.18	−0.93	−1.35, −0.51	0.59	0.10, 1.09	<0.001	<0.001	<0.001	<0.001
ΔWC	−4.34	−6.24, −2.45	−0.61	−1.73, 0.50	3.67	2.41, 4.92	<0.001	0.003	<0.001	<0.001
ΔSBP	−4.73	−9.97, 0.50	2.65	−0.66, 5.97	5.67	1.90, 9.44	0.008	0.061	0.006	0.714
ΔDBP	−5.22	−8.78, −1.66	1.33	−0.93, 3.60	4.04	1.48, 6.60	<0.001	0.008	<0.001	0.361
ΔSMI_wt	1.80	1.22, 2.38	0.33	−0.04, 0.71	−0.39	−0.82, 0.03	<0.001	<0.001	<0.001	0.035
ΔFat%	−3.47	−5.31, −1.63	−0.97	−2.16, 0.23	0.37	-0.97, 1.71	0.005	0.077	0.004	0.435
ΔALT	−41.92	−54.05, −29.79	−27.71	−35.30, −20.12	−13.29	−22.22, −4.37	0.001	0.156	0.001	0.050
ΔGGT	−22.40	−34.99, −9.81	−17.55	−25.42, −9.67	1.11	−8.31, 10.53	0.003	1.000	0.011	0.010
ΔGlucose	−7.17	−13.16, −1.18	−1.68	−5.37, 2.01	0.80	−3.52, 5.12	0.109	0.382	0.107	1.000
ΔInsulin	−7.71	−10.22, −5.20	−0.38	−2.03, 1.26	1.89	0.00, 3.78	<0.001	<0.001	<0.001	0.226
ΔHOMA–IR	−2.07	−2.82, −1.32	−0.01	−0.49, 0.47	0.58	0.03, 1.13	<0.001	<0.001	<0.001	0.343
ΔTG	−28.50	−51.01, −6.00	−6.41	−20.28, 7.46	7.44	−8.96, 23.83	0.043	0.305	0.037	0.616
ΔHDL	7.78	4.76, 10.81	3.84	1.94, 5.74	3.02	0.87, 5.18	0.038	0.094	0.039	1.000
ΔLDL	−4.32	−16.13, 7.48	−6.23	−13.60, 1.14	−8.59	−17.13, −0.05	0.835	1.000	1.000	1.000

ΔFAST	−0.23	−0.29, −0.17	−0.13	−0.16, −0.09	−0.07	−0.11, −0.03	<0.001	0.010	<0.001	0.148
ΔLSM	−2.09	−2.70, −1.48	−1.61	−1.99, −1.23	−1.11	−1.56, −0.66	0.035	0.576	0.035	0.282

NOTE. *P* value for post-hoc test was obtained through the Bonferroni method.

Abbreviations: CI, confidence interval; Bwt, body weight; BMI, body mass index; WC, waist circumference; SBP, systolic blood pressure; DBP, diastolic blood pressure; SMI_wt, weight-adjusted skeletal muscle index; fat%, fat percentage; ALT, alanine aminotransferase; GGT, γ-glutamyl transpeptidase; HOMA-IR, homeostasis model assessment of insulin resistance; TG, triglyceride; HDL, high-density lipoprotein; LDL, low-density lipoprotein; FAST, Fibroscan-aspartate aminotransferase; LSM, liver stiffness measurement

Table S3. Mediation analysis of anthropometric and clinical parameters in the relationship between weight change during follow-up and FAST score ≤0.35 at follow-up among subjects with baseline level >0.35.

Mediator	ΔWeight → Mediator			Mediator → Follow-up FAST≤0.35			ΔWeight → Follow-up FAST≤0.35		
	beta coefficient	95% CI	*P*	beta coefficient	95% CI	*P*	beta coefficient	95% CI	*P*
ΔGGT	1.347	0.298, 2.395	0.012	−0.010	−0.032, 0.012	0.383	−0.126	−0.270, 0.018	0.086
ΔeGFR	0.092	−0.611, 0.796	0.794	−0.010	−0.043, 0.022	0.537	−0.130	−0.271, 0.008	0.066
ΔHOMA-IR	0.303	0.216, 0.391	<0.001	0.391	−0.052, 0.835	0.083	−0.293	−0.566, −0.021	0.034
ΔALT	3.154	1.283, 5.026	0.001	−0.083	−0.127, -0.039	<0.001	0.037	−0.218, 0.294	0.771
ΔBMI	0.393	0.333, 0.452	<0.001	0.131	−0.240, 0.504	0.487	−0.199	−0.393, −0.006	0.042
ΔSMI_wt	−0.237	−0.306,−0.168	<0.001	−0.114	−0.634, 0.406	0.667	−0.138	−0.329, 0.052	0.154
ΔLDL	−0.167	−1.723, 1.387	0.830	−0.017	−0.033, −0.001	0.038	−0.161	−0.312, −0.011	0.035
ΔInsulin	1.118	0.846, 1.390	<0.001	0.048	−0.077, 0.174	0.448	−0.225	−0.484, 0.033	0.087
ΔWC	0.797	0.600, 0.994	<0.001	−0.078	−0.237, 0.081	0.337	−0.049	−0.253, 0.155	0.638
ΔGlucose	0.628	−0.190, 1.446	0.130	0.003	−0.025, 0.031	0.835	−0.151	−0.296, −0.006	0.041
ΔTG	2.268	−0.808, 5.344	0.145	−0.0004	−0.007, 0.007	0.911	−0.147	−0.290, -0.003	0.044
ΔHDL	−0.181	−0.551, 0.188	0.331	0.022	−0.044, 0.088	0.516	−0.117	−0.254, 0.019	0.092
ΔSBP	0.456	−0.206, 1.119	0.173	0.001	−0.037, 0.039	0.944	−0.115	−0.255, 0.024	0.106

ΔDBP	0.251	−0.191, 0.695	0.260	−0.019	−-0.075, 0.037	0.506	−0.103	−0.232, 0.025	0.114
ΔFat%	0.459	0.191, 0.727	0.001	−0.016	−0.153, 0.121	0.818	−0.084	−0.237, 0.068	0.277

NOTE. The indirect effect by ΔALT was significant in the relationship between weight change and follow-up FAST score <0.35 (indirect effect= −0.264, 95% CI −0.882, −0.124) from 1000 bootstraps.

Abbreviations: CI, confidence interval; GGT, γ-glutamyl transpeptidase; eGFR, estimated glomerular filtration rate; HOMA-IR, homeostasis model assessment of insulin resistance; ALT, alanine aminotransferase; BMI, body mass index; SMI_wt, weight-adjusted skeletal muscle index; LDL, low-density lipoprotein; WC, waist circumference; TG, triglyceride; HDL, high-density lipoprotein; SBP, systolic blood pressure; DBP, diastolic blood pressure; fat%, fat percentage

Figure S1. Area under the receiver operating characteristic curve of the multiomics-based model for the prediction of FAST score >0.35.

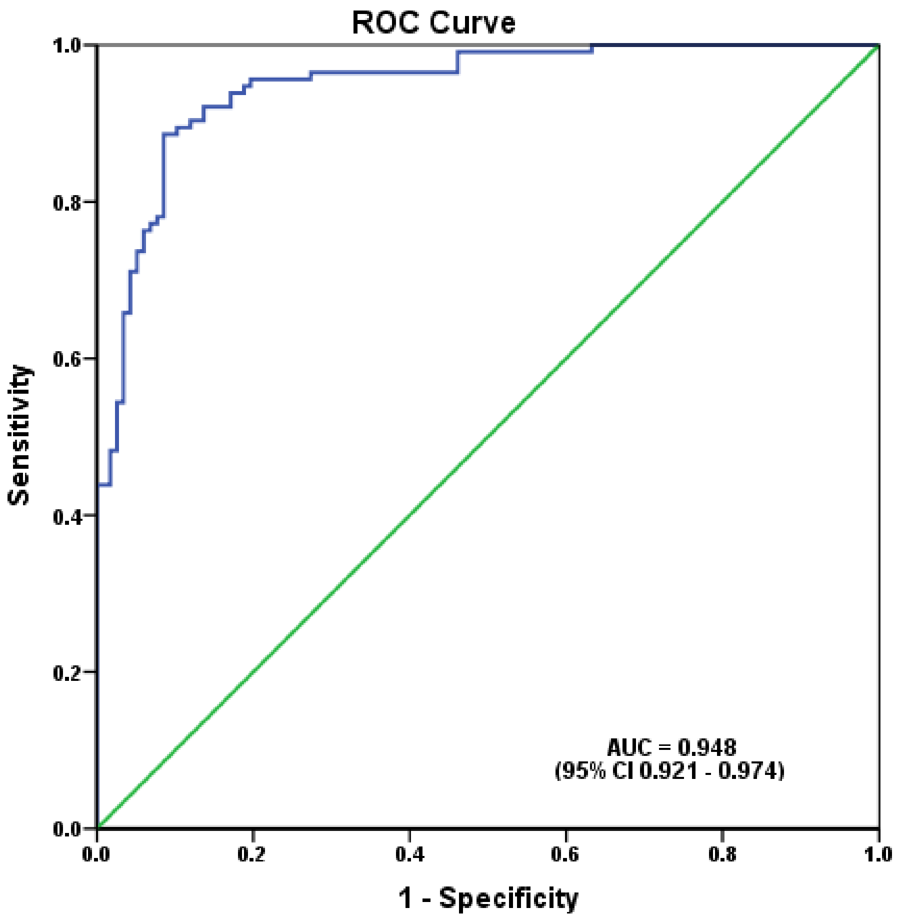

Supplement: Supplementary file 1 [file metabolites-13-00444-s001.zip › metabolites-2258565-supplementary.pdf]
